# *Chlamydia trachomatis* samples testing falsely negative in the Aptima Combo 2 test in Finland, 2019

**DOI:** 10.2807/1560-7917.ES.2019.24.22.1900298

**Published:** 2019-05-30

**Authors:** Kaisu Rantakokko-Jalava, Kati Hokynar, Niina Hieta, Anniina Keskitalo, Pia Jokela, Anna Muotiala, T. Sakari Jokiranta, Rutta Kuusela, Hannu Sarkkinen, Janne Aittoniemi, Tytti Vuorinen, Antti J Hakanen, Mirja Puolakkainen

**Affiliations:** 1 Department of Clinical Microbiology, Turku University Hospital, Turku, Finland; 2 Venereal Diseases Outpatient Clinic, Turku University Hospital, Turku, Finland; 3 University of Turku, Turku, Turku, Finland; 4 Department of virology, University of Helsinki, Helsinki, Finland; 5 Department of Virology and Immunology, University of Helsinki and Helsinki University Hospital, Huslab, Helsinki, Finland; 6 United Medix Laboratories Ltd, Helsinki, Finland; 7 Synlab Finland Ltd, Helsinki, Finland; 8 Satadiag, Pori, Finland; 9 Fimlab, Lahti, Finland; 10 Fimlab Laboratories, Tampere, Finland

**Keywords:** Chlamydia trachomatis, variant, molecular diagnostics

## Abstract

Since February 2019, over 160 *Chlamydia trachomatis* (CT) cases testing negative or equivocal by Aptima Combo 2 (AC2) but positive by Aptima CT test run with Panther instruments occurred in Finland. The AC2 test targets chlamydial 23S rRNA while the CT test targets 16S rRNA. Sequencing of 10 strains revealed a nucleotide substitution in 23S rRNA. The significance of this for the failure of the AC2 test to detect the variant is not yet known.

In mid-February, a discrepancy was noticed in testing results for chlamydia in the Clinical Microbiology Laboratory of Turku University Hospital, Turku, Finland. A first-void urine (FVU) sample from a person in their 30s had been referred for analysis with a multiplex sexually transmitted infection (STI) assay (Allplex STI Essential, Seegene, Seoul, Korea) and was clearly positive for *Chlamydia trachomatis* (cycle threshold, Ct 25; cutoff for positivity being ≤ 40). A urethral swab specimen taken on the same day was negative by Aptima Combo 2 (AC2) test run in the Panther Instrument (Hologic, San Diego, California (CA), United States (US)). The patient had visited the clinic for STI twice in January, and FVU samples on both visits had tested negative in the AC2 test. Moreover, this patient had a chlamydia-positive partner who was tested in another area of the country, where the Abbott m2000 test (Abbott Park, Illinois, US) is used for screening for chlamydia and gonorrhoea.

In the AC2 assay, chlamydial 23S rRNA and/or gonococcal 16S rRNA are amplified by transcription-mediated amplification reaction (TMA), and DNA probes with chemiluminescent label are used to detect the amplicons. The results are given in relative light units (RLU). The amplification of *C. trachomatis* and gonococci can be distinguished by different kinetic profiles. The cutoffs given by the manufacturer are RLU 25 for equivocal (RLU ≥ 25 – < 100) and 100 for a positive result (RLU ≥ 100) if only a chlamydial signal is detected. If both chlamydial and gonococcal signals are present or chlamydial result is indeterminate, the cutoff for *C. trachomatis* equivocal is 85 RLU (RLU ≥ 85 – < 250). Samples with ≥ 250 RLU are positive for *C. trachomatis*, if both chlamydial and gonococcal signals are present [1]. As the assay relies on automated equipment (Panther), when a given sample has been processed, the instrument flags the sample as negative, equivocal or positive for chlamydia and/or gonococcus. While the manufacturer’s thresholds are accounted for during flagging, the instrument may also take into account other parameters so the results displayed by the instrument may not always reflect an interpretation strictly based on the RLU.

The FVU sample positive in Allplex was tested by AC2 in duplicate and yielded RLUs 25 and 26, interpreted by the instrument as negative for both chlamydia and gonococcus. It was agreed that if there were other patients who had samples flagged negative for *C. trachomatis* despite of clinical suspicion, their samples should be tested also with the multiplex STI assay. During the next week, two such patients were detected (with Allplex test Cts 23 and 35 and AC2 RLUs 23 and 28, respectively).

As soon as the discrepancy was observed, the distributor of the AC2 test (Immunodiagnostic, Hämeenlinna, Finland) rapidly provided us with the Aptima CT (ACT) test kits. The ACT test targets chlamydial 16S rRNA and is recommended by US Centers for Disease Control and Prevention (CDC) guidelines for testing of *C. trachomatis* alone and/or for confirmation of the AC2 test results with an alternative gene target [2]. Since the vast majority of the samples in general yields RLUs < 10, we decided to retest all samples with RLU ≥10 with the ACT test since mid-February. The first three AC2 false negative samples (respectively from the three patients discussed above) were clearly positive with the ACT test with RLUs > 6,000 (cutoffs for low positive 100 and positive 5,000 [3]). During the first week of March, six more false-negative/equivocal patients by AC2 were found positive by ACT. 

## Case definition for false negative *Chlamydia trachomatis* and detected cases

A case was preliminarily defined as a person with an AC2-negative or equivocal sample yielding RLU in the range of 20–84 (with RLU < 25 being negative) and a positive result in the ACT test.

Between February and the end April 2019, a total of 26 samples from 25 patients fulfilling these criteria were identified in Turku. Of these, 11 were male FVUs, 12 female FVUs and two cervical swabs. The mean age of patients was 28 years (range: 17–48). Twenty-two samples were flagged in AC2 as negative for *C. trachomatis* and four as equivocal, three of the latter being requested new samples from patients whose samples had been flagged negative with RLUs 24–34 about 4 weeks earlier. Eighteen of the 26 samples were also tested with Abbott m2000 that amplifies two targets in the cryptic plasmid. All except one were positive (the one sample with Ct 35 in Seegene Allplex test remained negative).

## Prevalence of missed *Chlamydia trachomatis* cases in Southwest Finland

To assess the prevalence of AC2-negative or equivocal (RLU 20–84)/ACT-positive samples in Turku area (hospital district of Southwest Finland), a total of 943 samples sent for chlamydia testing with negative or equivocal result in the AC2 test were reanalysed by ACT. The percentage of ACT-positive results among the samples with various RLUs in the AC2 test is shown in Table 1.

**Table 1:** Samples negative or equivocal for *Chlamydia trachomatis* by Aptima Combo 2 retested by Aptima CT in Turku, Finland, 11–29 March 2019 (n = 943)

Qualitative result in the original AC2 test according to the instrument display	RLU in the original AC2 test	Number of samples tested by ACT test	Positive in the ACT n %		The AC2 test RLU values in the ACT positives
Negative or equivocal for CT and negative for GC	≤ 10	916	1	0.1	3
	11–15	9	1	11	15
	16–19	0	0	0	n.a.
	20–84	15	13	87	20–49^a^
Negative for CT and positive for GC	> 1,300	3	0	0	n.a.

AC2: Aptima Combo 2; ACT: Aptima CT; CT: *Chlamydia trachomatis*; GC: *Neisseria gonorrhoeae*; n.a.: not applicable; RLU: relative light units.

^a^ One sample with RLU 49 was flagged as *C. trachomatis* equivocal; the others were reported as negative by the Panther instrument.

Among all *C. trachomatis* positive samples by ACT or AC2 or both during the same period, 15% (15/98) were positive only with the ACT test, including one that was flagged as equivocal in AC2 test. Four of the AC2 negative (n = 3) or equivocal (n = 1)/ACT-positive samples were from patients who had been recalled for a control specimen because of a suspicious AC2 test results in early 2019. Without this known catch-up diagnostics the AC2-false negative samples would comprise 10% (10/98) of all chlamydia positives during those three weeks.

Samples referred for chlamydia-gonorrhoea screening are routinely not stored in Turku University laboratory. However, 17 samples (7 male and 6 female FVUs, as well as 2 vaginal and 2 cervical swabs) that had been sent for analysis with the previously used Anyplex STI-5 genitourinary panel and tested positive for one of those analytes (*Mycoplasma genitalium*, *M. hominis*, *Ureaplasma urealyticum*, *U. parvum* or *Trichomonas vaginalis*) were available. The samples were dated from June to December 2018. One male FVU sample taken in late June 2018 was AC2-negative (RLUs of aliquots 22 and 19) but ACT-positive. The patient had previously given a FVU sample in mid-June and its RLU value in the original AC2 run was 31 and flagged as negative.

Since samples with RLUs in the range of 20–84 (AC2 test) were almost always ACT-positive, their proportion of the studied samples was used as an approximation of the prevalence of AC2 negative or equivocal/ACT positive cases. During the latter half of 2018, their average proportion was 0.4% (38/9,472) of all tested samples and 6% (38/624) of samples reported as positive for *C. trachomatis *by AC2.

## Geographical distribution of cases

In addition to Turku University Hospital, the AC2 test is used in five other laboratories in Finland. HUSLAB, the largest clinical microbiology laboratory in Finland, serves the hospital district of Helsinki and Uusimaa and hospital district of Kymenlaakso. United Medix Laboratories Ltd and Synlab Finland Ltd provide chlamydia/gonorrhoea tests mainly for private sector clients and Finnish Student Health Service organisation operating in whole Finland but also for several cities in southern and western Finland. Furthermore, the AC2 test is used in local laboratories of the hospital districts of Satakunta and Päijät-Häme. Together these laboratories cover ca 50% of all chlamydia-gonorrhoea screening performed in Finland. 

After the finding of AC2 negative or equivocal/ACT-positive cases, the ACT test was introduced also in these laboratories to enable testing of samples giving RLU values above the background level in the AC2 test as well as to make possible targeted retesting of e.g. samples collected in the STI clinics. The number of samples tested and the numbers of AC2- flagged negative or equivocal/ACT-positive samples in HUSLAB and United Medix Laboratories are presented in Tables 2 and 3. In the local laboratories of Satakunta and Päijät-Häme hospital districts, seven and five ACT positive specimens with RLUs 20–40 and 13–25 in the original AC2 test, respectively, have been detected. Synlab Finland Ltd has detected 13 cases. The location of the hospital districts where cases have been found and the number of cases in each district is shown in the Figure.

**Table 2:** Samples re-analysed with Aptima CT test in HUSLAB, Finland, 6 March–30 April 2019 (n = 757)

Qualitative result in the original AC2 test according to the instrument display	RLU in the original AC2 test	Number of samples tested by ACT test	Positive in the ACT n %		The AC2 test RLU values in the ACT positives
Negative or equivocal for CT and negative for GC	≤ 10	330	2	0.6	5–7
	11–15	266	7	2.6	14–15
	16–19	71	13	18	16–19
	20–84	73	68^a^	93	20–46
	85–250	3	3^b^	100	89–97
Negative for CT and positive for GC	48–1,492	14	1	7.1	1,492

AC2: Aptima Combo 2; ACT: Aptima CT; CT: *Chlamydia trachomatis*; GC: *Neisseria gonorrhoeae*; RLU: relative light units.

^a ^Of 50 samples ≥ 25 RLU, one sample was flagged as equivocal in the original AC2 test.

^b^ Of three samples with RLU between 85 and 99, three samples were flagged as equivocal in the original AC2 test.

**Table 3:** Samples re-analysed with Aptima CT test in United Medix Laboratories Ltd, Finland, 1 March–7 May 2019 (n = 193)

RLU in the original AC2 test	Number of samples tested by ACT	Positive in the ACT n %		The AC2 test RLU values in the ACT positives
≤ 10	51	0	0	n.a.
11–15	101	2	2.0	13, 14
16–20	10	3	30	17, 18, 20
21–84	31^a^	31	100	21–33
85–250	0	0	0	n.a.

AC2: Aptima Combo 2; ACT: Aptima *Chlamydia trachomatis*; n.a.: not applicable; RLU: relative light units.

^a ^All were flagged as negative by the Panther instrument.

**Figure:**
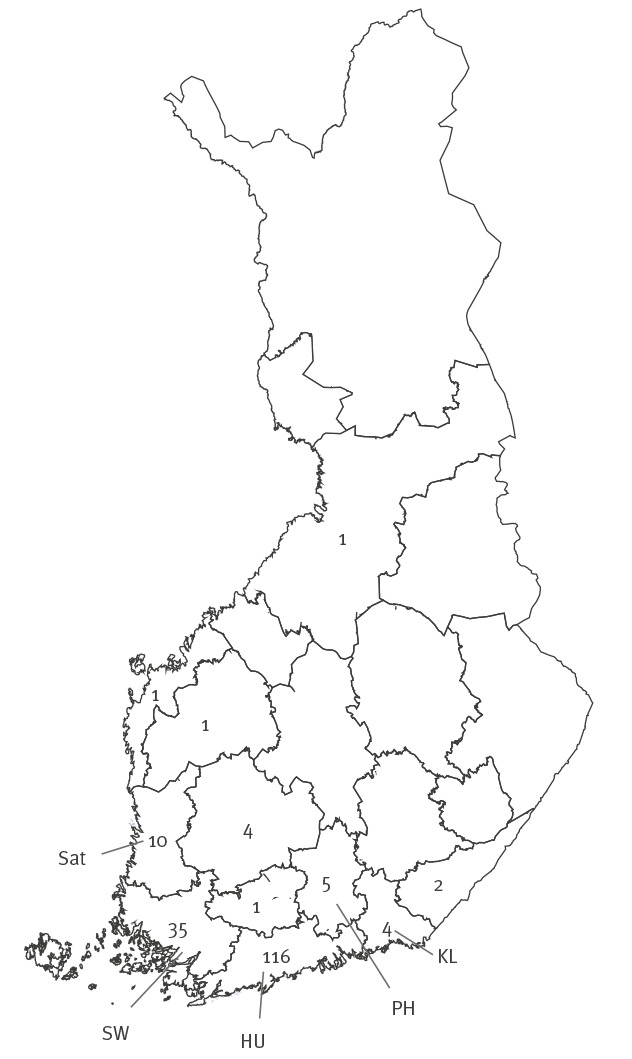
Numbers of detected AC2-negative or equivocal (RLU 20–84)/ACT-positive cases in hospital districts of Finland, mid-February–April 2019 (n = 180)

AC2: Aptima Combo 2; ACT: Aptima CT; HU: Helsinki and Uusimaa; KL: Kymenlaakso; PH: Päijät-Häme; RLU: relative light units; Sat: Satakunta; SW: Southwest Finland.

The number of cases found in each district figures for each district on the map. The districts in which both public and private laboratories use Aptima Combo 2 are indicated.

## Molecular analysis of cases

Respective samples from 10 cases, five originating from Turku and five from Helsinki area have been analysed at the University of Helsinki. Initial molecular characterisation (sequencing of 23S rRNA, 16S rRNA and typing based on *momp* gene) of the AC2 negative or equivocal (RLU 20–84)/ACT positive specimens show that all are genotype E, the most common genotype in Finland [4]. Compared with the reference sequence, *C. trachomatis* E/Bour (HE601870.1), there is a single nt change in the chlamydial 23S rRNA gene in position 1515 (C –> T) in the initially falsely negative or equivocal specimens. Thus far, we analysed only a limited number of AC2 negative or equivocal/ACT positive samples (n = 10). The sequences have been deposited in GenBank (GenBank accession number MN006991*). They all contain the same change, whereas this change cannot be found in the *C. trachomatis* reference strain sequences deposited in GenBank or in our previously sequenced *C. trachomatis* isolates [5] that had been AC2 positive.

## Discussion

Previously, failure of *C. trachomatis* nucleic acid detection test or tests to detect a variant strain was reported from Sweden. The Swedish new variant of *C. trachomatis* (nvCT) was first described in late 2006 and detected when assessing the reason behind an unexpected 25% decrease in the number of chlamydia cases in Halland area on the western coast of Sweden [6]. The strain had a 377-bp deletion within its cryptic plasmid that included the only targets of Roche and Abbott CT/GC tests of that time. The individuals with false negative test results remained untreated and unintentionally spread the infection further. The failure of diagnostics had provided the nvCT with a selective advantage, so that by the time of its discovery, nvCt had gained a high proportion (up to 64%) of all chlamydia cases in some areas where it could spread undetected [7,8]. It was estimated that the variant had emerged in 2000–2002, i.e. the spread could have happened in about 5 years by introduction of the strain into high-frequency transmitting populations [8].

Thus far, we have not been able to confirm that the change detected in the 23S rDNA is the reason behind the current phenomenon, however it remains a likely explanation. Specific primers and probes are used in the target capture, TMA, and detection steps of theAC2 assay. Even a single change in a suitable location could ruin performance of the test. The manufacturer has excluded erroneous function of the instruments and possibility of improperly functioning lots. A part of discrepant results may be due to higher sensitivity of single target detection in Aptima CT as compared with AC2 according to the performance data in the package inserts or lower sensitivity of the AC2 test in female FVU samples [1,3,9]. We do not know which of the patients (if any) detected thus far belong to same transmission chains. It is possible that strains with this change have been present in low numbers for a longer period of time.

Whatever the reason behind the false negative AC2 test results, the finding of 6–10% more positives by an alternative test reminds us of the general principles in chlamydia testing: no single test detects all cases and the sensitivity of a given method vary according to the sample type. However, also the lessons learned from the Swedish variant are valid and worth a reminder [8]: a single target should not be trusted in molecular diagnostics of infections. In our case the extended STI panel fortunately worked as a backup and it probably resulted in the discovery of the variant in an early phase of its spread. The first discrepant case was detected by an alert microbiologist trainee in reporting extended panel samples which are handled more manually than the chlamydia screening samples studied by the highly automated instrument with negative results sent directly to laboratory information system. Also, the close connection between laboratory and STI clinic played a crucial role in the rapid investigation of the discrepancy observed. The traditional quality indicators of chlamydia tests include close follow-up of the percentage of positives, which has not decreased in our laboratories. However, manufacturers of molecular tests should develop algorithms to monitor events and trends in the data produced by their instrument, e.g. percentage of samples in the grey zone. The increased use of molecular systems and automated ‘black box automates’ challenges us to find new strategies to keep up with the microbial evolution and safeguard the continuous high quality of diagnostics.

## *Addendum

The GenBank accession number was added on 04 June 2019.
