# Addendum for Euro Surveill. 2019;24(22)

**DOI:** 10.2807/1560-7917.ES.2019.24.23.190606e

**Published:** 2019-06-06

**Authors:** Eurosurveillance editorial team

**Affiliations:** 1 European Centre for Disease Prevention and Control (ECDC), Stockholm, Sweden

In the article ‘*Chlamydia trachomatis* samples testing falsely negative in the Aptima Combo 2 test in Finland, 2019’ by K Rantakokko-Jalava et al., published on 30 May 2019 a GenBank accession number was added on 04 June 2019 at the request of the authors.

